# In-situ Raman spectroscopy to elucidate the influence of adsorption in graphene electrochemistry

**DOI:** 10.1038/srep45080

**Published:** 2017-03-24

**Authors:** Wesley T. E. van den Beld, Mathieu Odijk, René H. J. Vervuurt, Jan-Willem Weber, Ageeth A. Bol, Albert van den Berg, Jan C. T. Eijkel

**Affiliations:** 1 BIOS - Lab on a Chip group, MESA^+^ Institute for Nanotechnology and MIRA Institute for Biomedical Engineering and Technical Medicine, University of Twente, Enschede, P.O. box 217 7500AE, The Netherlands; 2 Plasma and Materials Processing Group, Department of Applied Physics, Eindhoven University of Technology, Eindhoven, PO Box 513, 5600MB, The Netherlands

## Abstract

Electrochemistry on graphene is of particular interest due to graphene’s high surface area, high electrical conductivity and low interfacial capacitance. Because the graphene Fermi level can be probed by its strong Raman signal, information on the graphene doping can be obtained which in turn can provide information on adsorbed atoms or molecules. For this paper, the adsorption analysis was successfully performed using three electroactive substances with different electrode interaction mechanisms: hexaammineruthenium(III) chloride (RuHex), ferrocenemethanol (FcMeOH) and potassium ferricyanide/potassium ferrocyanide (Fe(CN)_6_). The adsorption state was probed by analysing the G-peak position in the measured *in-situ* Raman spectrum during electrochemical experiments. We conclude that electrochemical Raman spectroscopy on graphene is a valuable tool to obtain *in-situ* information on adsorbed species on graphene, isolated from the rest of the electrochemical behaviour.

Graphene has received much attention during the last decade because of its unique properties such as mechanical strength and impermeability to gasses^1^,^2^,^3^,^4^,^5^. Also the electrochemistry of graphene and energy storage using graphene is particularly interesting^6^ because of its high surface area^7^, high conductivity^1^,^7^ and low interfacial capacitance^8^. Although graphene shows unique behaviour as electrochemical interface^9^, the mechanism behind many of its specific properties is still not fully understood^10^,^11^,^12^,^13^. Importantly, the graphene Fermi level can be probed by its strong Raman signal, giving information about the graphene doping that is potentially caused by adsorbed atoms or molecules^14^,^15^,^16^,^17^,^18^,^19^,^20^,^21^,^22^. In Raman spectroelectrochemistry experiments, electrical and spectroscopic measurements are performed simultaneously, which gives real-time information about the graphene Fermi level during the electrochemical experiment^19^,^23^,^24^. With this, a better understanding of adsorption at graphene surfaces can be acquired, which can be used to improve the performance of graphene for electrochemical applications and energy storage. As a potential application in electrochemical SERS for plasmon-driven catalyst, an electrode surface could be covered by graphene^25^,^26^,^27^.

Here we will exploit the technique of Raman spectroelectrochemistry to obtain information about, among others, the reaction kinetics during Faradaic reactions on the surface of graphene. Often the desorption phase is the rate-determining step in nano- and microfluidic systems, where mass transport by diffusion is relatively fast compared to large scale systems. E.g. in redox cycling experiments slow desorption results in a lower effective diffusion coefficient and thereby in reduced signal levels^28^. Our working hypothesis in this investigation is that adsorption of redox-active species on graphene can be measured by Raman spectroscopy. From this hypothesis follows our research question: can we find evidence for the adsorption state during redox reactions at graphene in the Raman spectrum? To investigate this, we measured the Raman spectra of graphene during electrochemical measurements of three electroactive substances. We thereby chose substances of which, according to literature, the adsorption during electrochemical reactions differs. The results are obtained using chemical vapour deposited (CVD) graphene.

In the pathway of electrochemical reactions several steps can be distinguished as shown in Fig. 1. In simple model reactions and at low overpotentials the electron transfer is often the rate limiting step, while at higher overpotentials mass transport becomes the limiting factor^29^. The electrochemical activity is measured using cyclic voltammetry that reveals, for a reversible redox couple, an oxidation and a reduction peak. The distance between these peaks defines the peak potential separation. The Butler-Volmer equation describes the electrical current density upon application of an electrode potential. In this equation the standard rate constant is a measure of the kinetic facility and sets the sluggishness of the reaction^29^. A simulation was performed to determine the relation between the standard rate constant to the peak potential separation using the Nernst-Planck and Butler-Volmer equations. In the result, shown in Fig. S1, the logarithmic relation is shown for low standard rate constants and levels off to the theoretical value of 59 mV for higher standard rate constants.

To explain redox reactions for complex molecules two mechanisms are recognized: inner-sphere and outer-sphere electrode reactions^30^. In outer-sphere electrode reactions the reactant and the product do not require strong interaction with the electrode surface and therefore do not require ad- and desorption (step 2 in Fig. 1)^29^. Outer-sphere redox molecules that we will use are RuHex (hexaammineruthenium(III) chloride)^12^,^29^ and FcMeOH (ferrocenemethanol)^10^. Although FcMeOH is an outer-sphere redox molecule, it is known to adsorb (mostly) reversibly to the graphene surface with a desorption time constant of several minutes^10^,^31^. After performing electrochemical experiments with FcMeOH a fraction of ~1% of a monolayer has been found to permanently bound to the graphene surface, most probable at local defects sites for which the exact mechanism is unidentified^10^,^32^. In contrast to outer-sphere molecules, species involved in inner-sphere electrode reactions require a strong interaction with the electrode surface in order to exchange electrons. Hereby the adsorption mechanism could be specific for an electrode material^29^. In our investigation, the tested inner-sphere redox couple is Fe(CN)_6_ (potassium ferricyanide/potassium ferrocyanide), which is known to have different reaction kinetics on monolayer graphene and pyrolytic graphite^12^. For further interpretation of the voltammogram also the overpotential is of importance. For the redox molecules in our experiments (assuming symmetrical charge transfer coefficients), the distance between the oxidation and reduction peak in the voltammograms represents the peak separation voltage required for the reversible reaction to occur at the specific electrode^29^.

Whenever molecules adsorb to the graphene surface, the graphene Fermi level is shifted by local doping (changing the carrier concentration), which can be probed by Raman spectroscopy^19^,^33^. The measured Raman spectrum *S*_m_ will then be a superposition of two spectra: the graphene with adsorbed molecules *S*_ad_ and the bare graphene *S*_gr_. For the ratio between both we define an occupation factor *β* as follows


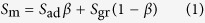


when this factor is low β ≈ 0 solely the bare graphene spectrum is measured, indicating no interaction between the graphene and the molecules in solution. For a large factor β ≈ 1 the graphene is entirely occupied by adsorbed molecules which interact with the graphene and therefore change the Raman spectrum.

A typical Raman spectrum measured for our graphene is shown in Fig. 2. In this measured Raman spectrum the G-peak position (in cm^−1^) is analysed, since this is the most sensitive indicator for the graphene Fermi level and therefore the adsorption level^8^,^19^. When doping is induced in the graphene, this will cause two effects in the G-peak position curve. Firstly, doping will lead to a shift in the charge neutrality point voltage V_CNP_ , which is the applied gate voltage corresponding to a minimum in the G-peak position curve, probing the graphene Fermi level. This will cause the valley to move horizontally (n-doping to more negative voltages, p-doping to more positive)^14^. Secondly, the G-peak is known to shift position upon electrochemical doping^8^, resulting in a vertical shift of the curve. In addition to these two effects, the slope of this G-peak position curve is electrolyte concentration dependent: at lower ionic concentrations this slope is decreasing whenever species adsorb to the surface, indicating a decrease of electron and hole mobility^14^.

Finally, in literature we find that at defects in the graphene, such as grain boundaries, the electron exchange rate is locally orders of magnitude higher^10^,^11^,^34^,^35^. When interpreting the measurement results we will also hypothesize that two types of reaction sites are present at the graphene: at the basal plane (high overpotential, reversible bonding) and local defect sites (low overpotential, almost permanent bonding). The redox reaction kinetics for graphene thus are complex, since the ability for electron exchange is not uniform over the surface. This reaction model is schematically depicted in Fig. 3. In literature we find that the reaction rates of the outer-sphere reactions (that not depend on adsorption) mainly depend on the availability of the more reactive sites^12^. In the *in-situ* Raman spectroscopy experiments the adsorption (and possibly the approach) of redox species can be probed independently of the remainder of the electrochemical behaviour. To explain the electrochemical voltammogram data, we will thus distinguish adsorption and electron exchange at reactive sites (Fig. 3a–d) from adsorption and electron exchange (with a different overpotential) at the basal plane (Fig. 3e–h).

## Results and Discussion

To test our hypotheses, the adsorption of the three redox active species during electrochemical experiments using *in-situ* Raman spectroscopy was studied by measuring on two graphene electrode devices. After measuring the baseline in background electrolyte, RuHex and FcMeOH were measured with the first graphene device. Subsequently, Fe(CN)_6_ was measured using the second graphene device.

### RuHex

**Figure 4:**
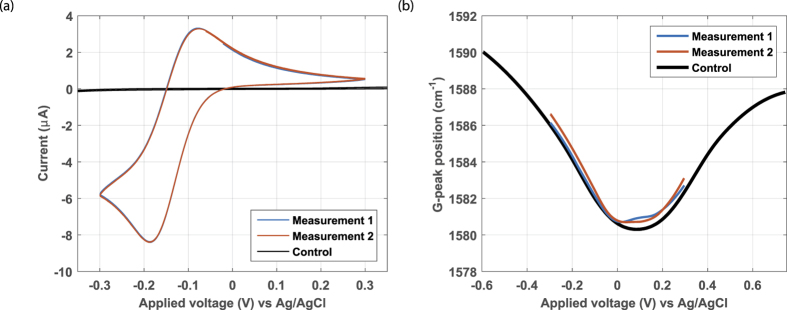
Raman spectroelectrochemistry of RuHex at graphene electrode device 1. The voltammogram (**a**) shows two performed RuHex measurements and the control curve of the background electrolyte (black). The G-peak position extracted from the *in-situ* recorded Raman spectrum vs the applied potential (**b**) shows curves very similar to the control (black).

The cyclic voltammogram of RuHex at the graphene electrode is shown in Fig. 4a. The profile is in accordance with RuHex voltammograms at graphene electrodes found in literature^12^. Furthermore, the peak separation of 0.10 V is close to the theoretical value of 59 mV where the deviation is explained by an IR-drop in the solution. As shown in Fig. S1, a low peak separation voltage is related to a large standard rate constant *k*^0^, therefore we conclude that the electrode kinetics of RuHex are such that it is a fast reaction^29^. In Fig. 4b the *in-situ* recorded G-peak position curves are displayed, which are at about the same position as the baseline curve. Both curves are constant over several measurements, showing no change over time. This dataset indicates that RuHex adsorption to the graphene electrode was absent or below our detection limit (β ≈ 0). As expected for an outer-sphere redox couple that does not need adsorption to react, a sizeable redox current was still observed despite this lack of adsorption.

### FcMeOH

**Figure 5:**
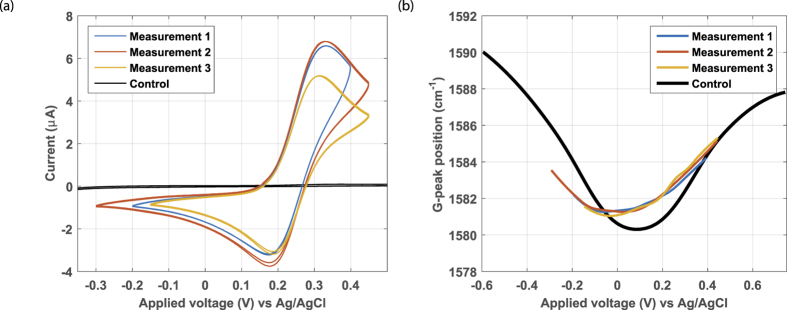
Raman spectroelectrochemistry of FcMeOH at graphene electrode device 1. The voltammogram (**a**) shows three performed FcMeOH measurements and the control curve of the background electrolyte (black). The G-peak position extracted from the *in-situ* recorded Raman spectrum vs the applied potential (**b**) shows curves different from the control (black), indicating adsorption.

The observations differ when the measurement is repeated for FcMeOH, as shown in Fig. 5. The larger current in the voltammogram could indicate that the graphene of device 2 contains more reactive sites than the graphene of device 1. The peak separation voltage is larger for FcMeOH (0.14 V, see Fig. 5a) than for RuHex (0.10 V, see Fig. 4a), indicating a lower standard rate constant *k*^0^ (see Fig. S1) and thus slower electrochemical kinetics^29^. In the G-peak position curve (Fig. 5b) the charge neutrality point (the valley) has shifted from about 0.1 *V* to about 0.0 *V*. The peak also red-shifted (higher G-peak position). Both effects can be explained by the adsorption of FcMeOH molecules. Furthermore, the valley (Fig. 5b) is wider than the baseline curve indicating a decrease in carrier mobility^14^. Based on these observations we conclude that FcMeOH adsorbs to the graphene surface. Adsorption indeed is also reported in literature^10^,^31^,^32^, with a relative large adsorption factor (β ≫ 0). Because the G-peak curve is already displaced to its final position at the first measurement, equilibration must be fast indicating a large adsorption rate constant of the FcMeOH.

### Fe(CN)_6_

**Figure 6:**
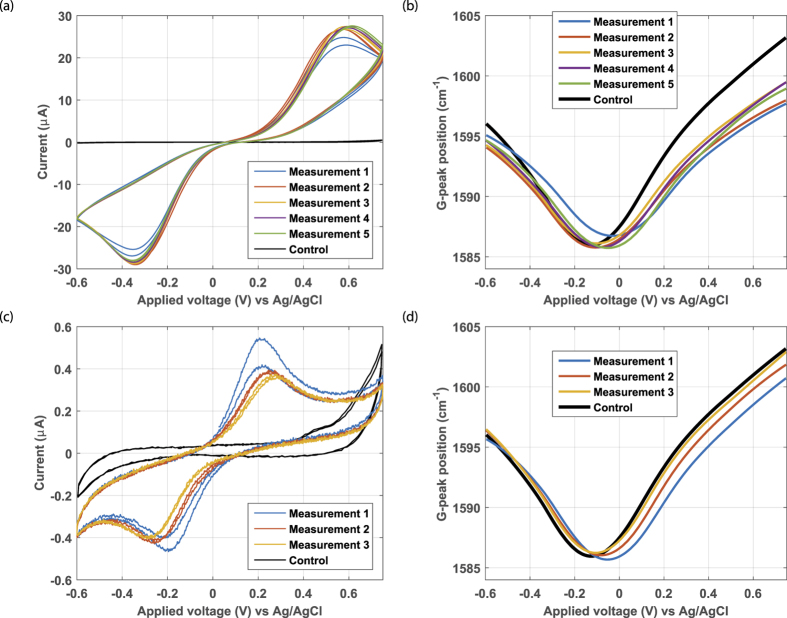
Raman spectroelectrochemistry of Fe(CN)_6_ at graphene electrode device 2 (measurement series 1, **a,b**) and subsequent Raman spectroelectrochemistry of background electrolyte (with absorbed Fe(CN)_6_) graphene electrode device 2 (measurement series 2, **c,d**) to test the desorption of Fe(CN)_6_. The voltammograms (**a,c**) show the results of a sequence of respectively five and three Fe(CN)_6_ measurements and the control baseline curve measured in the background electrolyte (black). The G-peak position extracted from the *in-situ* recorded Raman spectrum vs the applied potential (**b,d**) show curves different from the control (black), that respectively indicate adsorption and gradual recovery to the control curve (black).

The spectroelectrochemistry of Fe(CN)_6_ was tested on graphene device 2 resulting in the voltammogram shown in Fig. 6a. The peak separation voltage is much larger for Fe(CN)_6_ (approximately 0.9 V) than for RuHex (0.10 V) and FcMeOH (0.14 V), indicating a much lower standard rate constant *k*^0^ (see Fig. S1) and thus much more sluggish electrochemical kinetics. During the first five scans (during the first two measurements) the current is increasing every scan, while in the following two measurements the current peak height is constant. In these last two measurements however, the oxidation peak position is slightly shifting to a more positive voltage. This minor shift could be caused by the need for a higher overpotential for the reaction to take place by a (partially) changed surface. An alternative explanation for this small shift on top of the large peak separation voltage is that the graphene electrode resistance is increased (larger IR-drop) by the (local) doping effect of more adsorbed molecules, causing a lower graphene-solution voltage at the same applied device voltage whenever a Faradaic current is flowing. This local doping can be observed by the blue-shift (lower Raman shift) of the G-peak position at 0.6 V. The measured graphene sheet resistance multiplied by the measured current results in a voltage drop of about 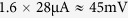, which is in the range of the observed peak shift in the voltammogram. Since the graphene sheet resistance is affected quite easily by a change in charge carriers, this is a plausible explanation.

In Fig. 6b the G-peak position curve is shown. During the first cycle a large horizontal shift is observed, but unexpectedly red-shifted (higher G-peak position). Our expectation is, that a shift to a more positive voltage is accompanied by a red shift. For the following measurements (2–5) the pattern is slightly different, the G-peak position valley is lower than in the first measurement and is slightly moving to the right with each measurement. Both observations can be explained by Fe(CN)_6_ adsorption, whereby the difference between the first and the subsequent measurements suggest that two adsorption states exist. In addition the slope becomes shallower. The observations are as expected for the inner-sphere redox couple Fe(CN)_6,_ that needs adsorption before reacting.

To test whether also evidence for desorption of Fe(CN)_6_ can be found, the reservoir was three times rinsed with background electrolyte and subsequently the measurements were performed. The results are shown in Fig. 6c,d after rinsing and without added Fe(CN)_6_. The voltammogram shows a quite different behaviour: the peak separation voltage has reduced from approximately 0.9 V to approximately 0.4 V and the current has decreased 50-fold. The decrease in peak separation voltage could be explained by a difference in standard rate constant resulting in different overpotentials. The voltammogram in Fig. 6c is quite similar to the one of Fe(CN)_6_ on graphite, where reaction takes place at reactive edge sites with low overpotential. The traces in Fig. 6c can then be explained by redox reactions at a low density of sites (possibly grain boundaries) with faster electrochemical kinetics at the graphene. The currents also remain stable after an initial decrease, indicating a very low desorption rate of this fraction of Fe(CN)_6_. In the first measurement the minimum in the G-peak position curve is still quite similar to the position in Fig. 6d, indicating still extensive adsorption, presumably at the basal plane. From literature it is known that species which adsorb to the electrode could cause a change of the peak potentials, heights and widths^29^,^36^. In the subsequent measurements of the G-peak position curve, a clear recovery towards the control state can be seen, indicating desorption, presumably from the basal plane. The voltammogram however does not entirely recover to the situation of the control experiment. The residual current could be caused by a fraction of defect-adsorbed Fe(CN)_6_ that is hard to desorb, similar to FcMeOH.

Finally, a measurement was performed to establish whether the signal shown in Fig. 6a,b could be recovered. Indeed the recovery of the Fe(CN)_6_ activity was verified in the Fe(CN)_6_ solution with the results as shown in Fig. S6. The voltammogram oxidation peak is located at a slightly higher voltage, which can be explained by the shift in the CNP of the graphene indicating the Fermi level to a higher value (0 V) than in Fig. 6a,b (−0.1 V). Alternatively, it could be caused by a further decreased graphene charge carrier concentration. In Section S2.3 the results of the graphene device 1 tested with Fe(CN)_6_ can be found, showing to what extent the device history influences its behaviour.

In the model, the occupation factor *β*, as visible from the Raman spectrum, and the amount of reacting species were directly coupled. The data for RuHex, an outer sphere redox substance, were not in conflict with this model, as for such substances redox activity is independent of adsorption. For FcMeOH, another outer sphere redox substance, adsorption was still found. This, however, is in accordance with literature reports, whereby it could not be established whether this adsorption contributes to the redox activity. The results for Fe(CN)_6_ were also in line with expectations, as we observed both adsorption and electroactivity for this inner sphere redox couple. We however observed in the Fe(CN)_6_ desorption experiments that two type of reactions occur, with low and high overpotential. The latter one has a major contribution to the current. The former one is persistent and remains when the solution is flushed.

A further complication in the analysis of the cyclic voltammograms is that the graphene conductivity is influenced by adsorbed species in two ways. Firstly, the adsorbed molecules on the basal plane (the smooth homogeneous graphene crystals) influence the conduction by changing the carrier concentration. For FcMeOH (uncharged) the charge neutrality point V_CNP_ shifted to a more negative voltage and for Fe(CN)_6_ (−3 and −4 charge) to a more positive voltage, suggesting that the charge of the molecule determines whether n- or p-doping is induced. Secondly, graphene electron mobility is reduced by grain boundaries (which can be visualized by mapping the intensity of the D-peak in the Raman spectrum)^37^,^38^. Species adsorbed on these grain boundaries could increase the conductivity^39^,^40^. For larger currents (I > ~ 1 μA) a lower conductivity affects the voltammogram by increasing the required overpotential (shifting the peaks to more extreme voltages) and lowering the current peak heights.

Evidence for the adsorption state during redox reactions at graphene in the Raman spectrum was found. The adsorption state was probed by analysing the G-peak position in the measured *in-situ* Raman spectrum during electrochemical experiments. Prior to every experiment a G-peak position curve was recorded for the bare graphene, serving as a baseline for subsequent measurements. For RuHex no change in the G-peak position curve was found during the electrochemical experiment compared to the baseline in solely background electrolyte, which was as expected. In case of FcMeOH a clear stable negative shift in the G-peak position curve was observed, indicating a strong adsorption state to the graphene, potentially caused by π-interactions or electrostatic interactions. For Fe(CN)_6_ a clear positive shift in the G-peak position curve could be seen, however for this species the measurement required some time to settle towards a constant voltammogram and G-peak position curve. This is suggesting that FcMeOH and Fe(CN)_6_ interact differently with the graphene. Furthermore, when measuring Fe(CN)_6_ on the graphene electrode after FcMeOH we found no clear difference in the voltammogram compared to Fe(CN)_6_, while the G-peak position curve indicated that both species were adsorbed.

Based on these findings we conclude that Raman spectroelectrochemistry on graphene is a powerful tool to obtain *in-situ* information on adsorbed species on graphene, independent of the cyclic voltammograms measured. This makes graphene an even more interesting electrode material for electrochemical sensing as it allows a fundamental investigation of detailed adsorption mechanisms by Raman spectrometry. We found indications that the adsorption of species for the larger part takes place on the less reactive basal plane, while local reactive sites mainly determine the electrochemical behaviour. We also observed additional effects of the adsorbed species to the graphene surface, where a slight change in the electrochemical behaviour is most probably caused by changes in graphene charge carrier density and passivation of the surface.

## Methods

The peak potential separation as function of the standard rate constant is simulated in COMSOL Multiphysics 5.1 using the ‘Transport of Diluted Species’ physic module. A round electrode is modelled in a cylindrical coordinate system with boundary condition: 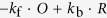, in a similar fashion as reported earlier in Odijk *et al*.^41^. The diffusion coefficient of the electrolyte is set to 7 × 10^−10^ m^2^/s. Current is calculated by integrating the normal flux at the electrode boundary over its area and multiplying by F. On t = 0 is the concentration of the reduced species 1 mM and the oxidized species are absent. The transient behaviour is simulated by scanning the overpotential between −1 V and 1 V with 25 mV/s.

The schematic of the fabrication and measurement setup of the graphene devices are shown in Fig. S2. Chemical vapour deposited (CVD) graphene synthesized on copper foil (Alfa Aesar no. 13382) is transferred to a silicon dioxide substrate using a traditional wet transfer procedure using poly(methyl methacrylate) (PMMA) as a supporting polymer. Two gold electrical contacts are deposited on this graphene layer using a steel shadow mask and subsequently wire bonded to a printed circuit board (PCB). The resistance between the contact pads was measured to be 1.6, which is indicating a good contact to the graphene. Subsequently, the gold pads and the wire bonds were covered by epoxy (hysol), leaving a graphene area for contacting the solution open of approximately 30 mm^2^ for both devices.

The setup used in this experiment as shown schematically in Fig. S2f and in Fig. S3. It allows for simultaneous recording of the Raman spectrum while performing electrochemical experiments. Electrical potentials and currents are applied and measured using a Biologic SP300 potentiostat, always at a scan rate of 25 mV/s with a low-pass filter (*f*_cutoff_ = 5 Hz). To the background electrolyte, 0.1 M KNO_3_, the reactive species (RuHex, FcMeOH and Fe(CN)_6_) were added in a concentration of 1 mM. Subsequently, 100 μ*L* of solution was applied to the graphene device. All potentials were measured against an Ag/AgCl reference electrode (WPI Dri-Ref-450). The spectra are recorded using a WITec alpha 300 system with a 532 nm laser at 1 mW with an immersion objective for measuring in liquid (Nikon Fluor 40x 0.8 W DIC). Analysis of the recorded Raman spectra is performed using a MATLAB script, which accounts for an offset in the spectrometer by fitting the Rayleigh peak. Next Lorentzian curves are fitted to the graphene peaks to obtain their positions and intensities. The voltammetric measurement protocol was as follows. In each measurement three scans were performed in series (whereby the results of the last two scans are displayed). Measurements in a series were performed with a time spacing of approximately 1 minute.

## Additional Information

**How to cite this article:** van den Beld, W. T. E. *et al*. In-situ Raman spectroscopy to elucidate the influence of adsorption in graphene electrochemistry. *Sci. Rep.* **7**, 45080; doi: 10.1038/srep45080 (2017).

**Publisher's note:** Springer Nature remains neutral with regard to jurisdictional claims in published maps and institutional affiliations.

## Supplementary Material

Supplementary Information

## Figures and Tables

**Figure 1:**
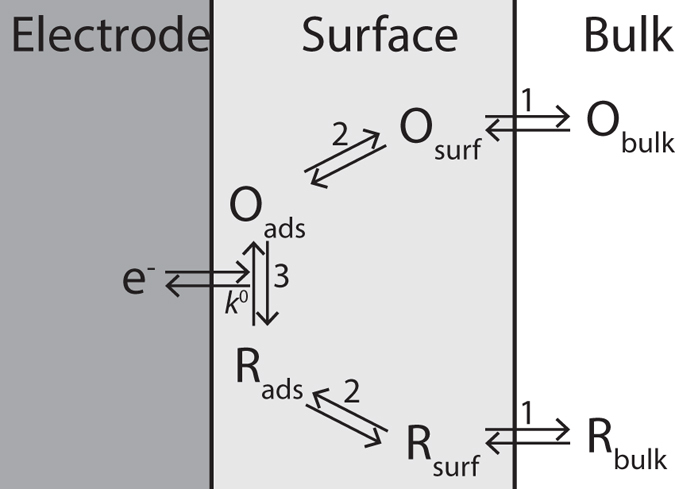
General pathway of an electrochemical reaction at an electrode surface that is composed of mass transfer towards the electrode surface (1), depending on the species ad- and desorption to the electrode (2) and electron exchange at the electrode (3)^29^.

**Figure 2:**
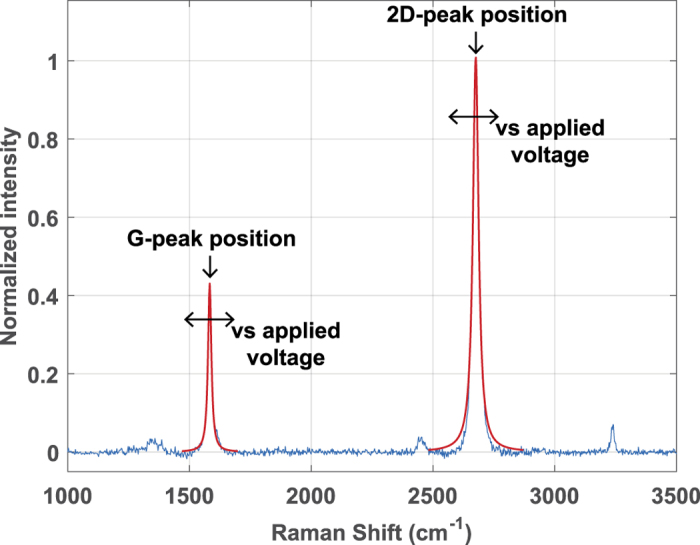
A typical measured Raman spectrum of our graphene revealing two distinct peaks. the G-peak and the 2D-peak. In the Raman spectroelectrochemistry experiments both peak positions are changing when electrode voltages are applied. In analysis of these experiments, the G-peak position is displayed next to the recorded cyclic voltammograms.

**Figure 3:**
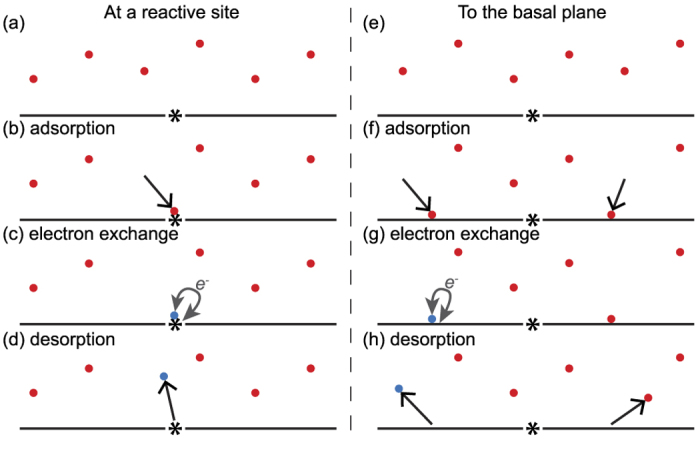
General schematic of redox active species adsorbing and optionally exchanging electrons at the graphene surface. The black line represents the graphene basal plane (the smooth homogeneous graphene crystals) and the *-symbol a grain boundary, edge or other site which has a higher reactive capability. The electron exchange rate is much lower at the basal plane than at the reactive sites, however the vast majority of the graphene surface consists out of graphene basal. In each of the two pathways a series of steps are drawn, in the pathway on the left: (**a**), **adsorption of a molecule** at a reactive graphene site (**b**), **electron exchange and adsorption** for a certain average time (**c**) and **desorption from the graphene surface** (**d**). In the pathway on the right (**e**), **adsorption of a molecule** to the graphene basal plane (less reactive) (**f**), **electron exchange** for some of the adsorption species **and adsorption** for a certain average time (**g**), and **desorption from the graphene surface** (**h**).
