# Supplementary material for: In-situ Raman spectroscopy to elucidate the influence of adsorption in graphene electrochemistry
Source: Sci Rep. 2017 Mar 24;7:45080. doi: 10.1038/srep45080 (PMC5364475; doi:10.1038/srep45080)
Supplement: Supplementary Information [file srep45080-s1.pdf]

In-situ Raman spectroscopy to elucidate the influence of adsorption in graphene electrochemistry

Wesley T.E. van den Beld[1,*], Mathieu Odijk[1], René H.J. Vervuurt[2], Jan-Willem Weber[2], Ageeth A. Bol[2], Albert van den Berg[1], and Jan C.T. Eijkel[1]

[1]BIOS - Lab on a Chip group, MESA+ Institute for Nanotechnology and MIRA Institute for Biomedical Engineering and Technical Medicine, University of Twente, Enschede, P.O. box 217 7500AE, The Netherlands.
[2]Plasma and Materials Processing Group, Department of Applied Physics, Eindhoven University of Technology, Eindhoven, PO Box 513, 5600MB, The Netherlands.
[*]w.t.e.vandenbeld@utwente.nl

Supplementary Information

S1 Theory

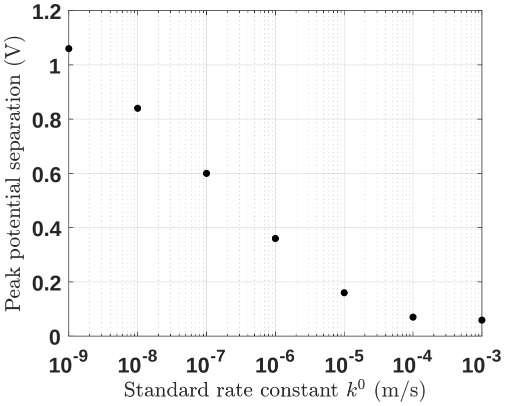

Figure S1. Simulation result showing the relation between the standard rate constant k^0 and the peak potential separation.

S2 Experimental

S2.1 Fabrication process

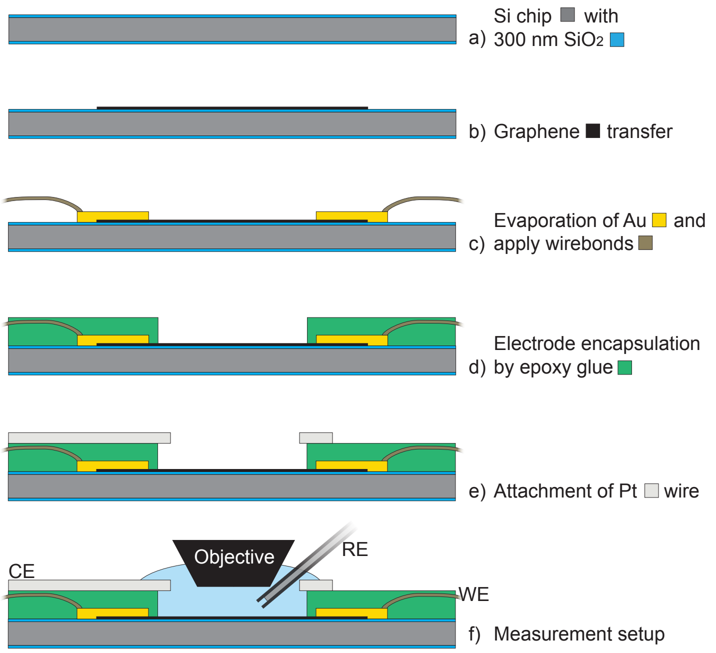

Figure S2. Schematic of the graphene device fabrication process for Raman spectroelectrochemistry. To a silicon substrate with 300 nm of silicon dioxide (a) graphene is transferred (b). Two gold contact pads are evaporated by e-beam evaporation (c), and encapsulated by epoxy glue (hysol) (d). Finally, a platinum wire is glued on top of the device (e). In the measurement setup, the wires bond to the graphene serve as the working electrode (WE), the platinum wire as the counter electrode (CE) and Ag/AgCl electrode as the reference electrode (RE), all contacting the liquid (light blue) (f). Using an immersion objective, the in-situ Raman spectra are recorded.

S2.2 Setup

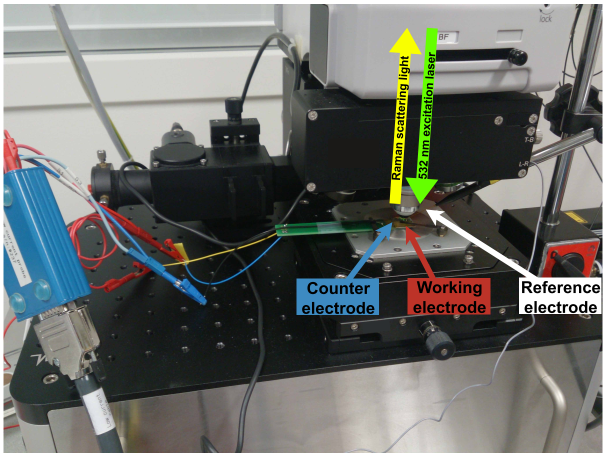

Figure S3. Photograph of the Raman spectroelectrochemistry setup showing the dipstick device connected to the potentiostat while located in the Raman microscope. The graphene layer served as working electrode, a platinum wire as counter electrode and a silver/silver chloride electrode as reference electrode (indicated in respectively red, blue and white). The Raman spectrum (yellow) is in-site recorded by using a 532 nm excitation laser (green).

S3 Additional results

To obtain an Raman spectroelectrochemistry baseline of the graphene electrode device 1 for the subsequent redox measurements, the Raman spectrum was recorded in solely the background electrolyte. Using MATLAB a smooth line (To all measurement points curves were fitted by the 'smooth' function with method 'rloess', which performs a local regression using linear least squares method (see also the MATLAB R2015b documentation: http://www.mathworks.com/help/matlab/).) was fitted to the data points to obtain the averaged baseline curve.

S3.1 Baseline measurements

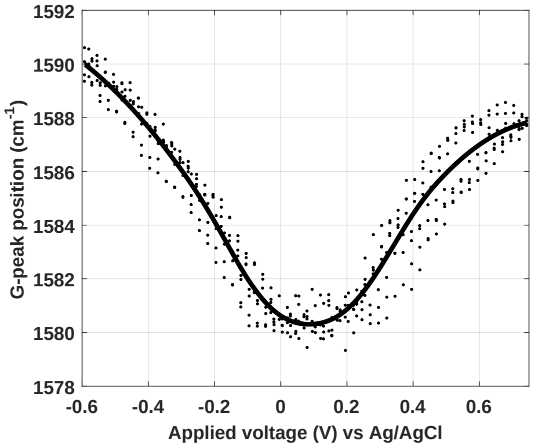

Figure S4. The G-peak position in the Raman spectrum as a function of the applied potential measured in background electrolyte at graphene electrode device 1, serving as a baseline for the subsequent measurements.

The data points with fitted baseline curve of device 1 is shown in Figure S4. The baseline shape and position correspond well to curves obtained in similar studies reported in literature.[1,2] Subsequent measurement results will be compared to this baseline measurement to monitor the change.

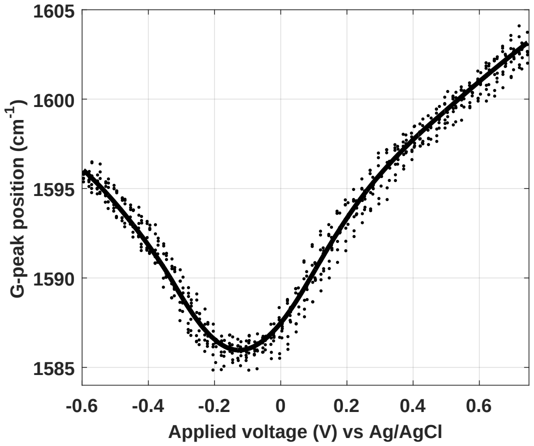

Figure S5. The G-peak position in the Raman spectrum as a function of the applied potential measured in background electrolyte at graphene electrode device 2, serving as a baseline for the subsequent measurements.

To have a clean graphene surface (without previously adsorbed molecules), we continued the redox measurements on a fresh device (device 2). Again a baseline curve was measured, which is shown in Figure S5. The measured baseline curve is different from the baseline curve of the previous device (Figure S4). This could be caused by a difference in PMMA residues on the graphene[3–5] and/or a different substrate interaction.[6] The shape of the curve is again as expected from literature.

S3.2 Electrolyte adsorption measurements

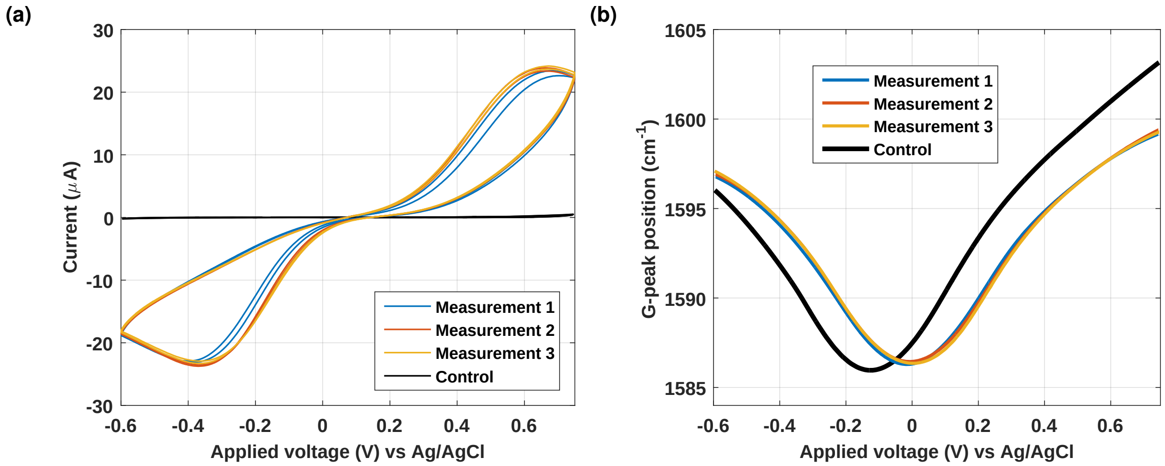

Figure S6. Raman spectroelectrochemistry of $Fe(CN)_6$ at graphene electrode device 2 (measurement series 3). The voltammogram (a) shows three performed $Fe(CN)_6$ measurements and the control curve (black) of solely the background electrolyte. The G-peak position extracted from the in-situ recorded Raman spectrum vs the applied potential (b) shows again a curve different from the control (black).

S3.3 $Fe(CN)_6$ after FcMeOH

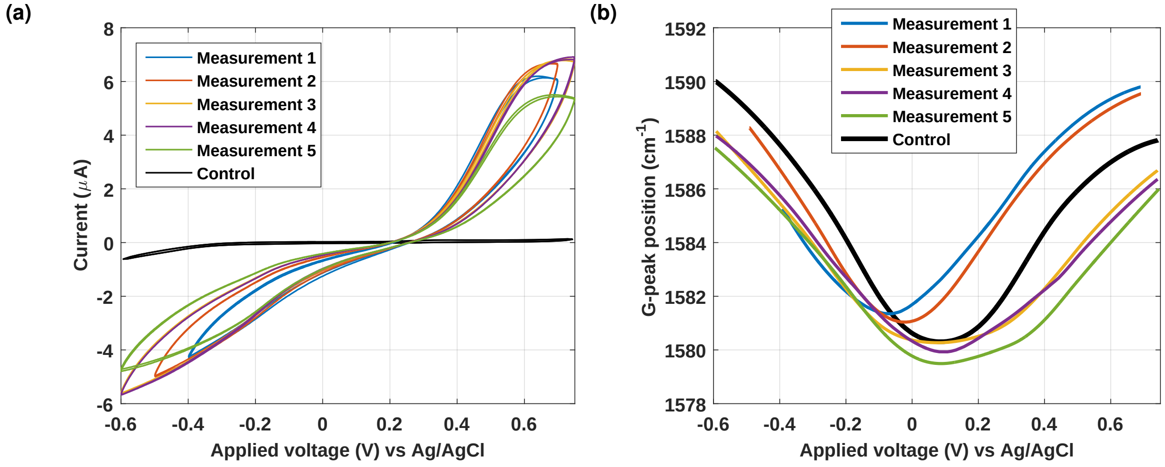

Figure S7. Raman spectroelectrochemistry of $Fe(CN)_6$ at graphene electrode device 1 (on which previously the adsorption of FcMeOH was tested). The voltammogram (a) shows five performed $Fe(CN)_6$ measurements and the control curve of the background electrolyte (black). The G-peak position extracted from the in-situ recorded Raman spectrum vs the applied potential (b) shows curves different from the control (black).

In addition to the preceding experiments, $Fe(CN)_6$ Raman spectroelectrochemistry was also applied to device 1 following the experiment with FcMeOH. In the resulting voltammogram shown in Figure S7a the redox peaks of FcMeOH appear absent and the $Fe(CN)_6$ reduction peak is shifted to a more negative voltage. The G-peak position curve in Figure S7b is revealing more on the details of adsorbed molecules. By comparing the curve to the case of FcMeOH in Figure 3b, we see that during the first two measurements the FcMeOH is still adsorbed on the graphene, judging from the shift in G-peak position relative to the bare graphene. In subsequent measurements the G-peak shifts to a position similar to the one observed in Figure 4 and S6, indicated that the $Fe(CN)_6$ is adsorbing to the graphene and potentially replaces a fraction of the adsorbed FcMeOH. In the final measurement 5 (green trace) a combination of both the FcMeOH and $Fe(CN)_6$ curves is found, thus indicating that both species are adsorbed to the graphene surface. Also in this case we therefore observe that there is no direct relation between FcMeOH adsorption and its electroactivity.

References

1. Das, A. *et al.* Monitoring dopants by raman scattering in an electrochemically top-gated graphene transistor. *Nature nanotechnology* **3**, 210–215 (2008).

2. Zhong, J.-H. *et al.* Interfacial capacitance of graphene: Correlated differential capacitance and in situ electrochemical raman spectroscopy study. *Electrochimica Acta* **110**, 754–761 (2013).

3. Chen, T.-Y. *et al.* Label-free detection of dna hybridization using transistors based on cvd grown graphene. *Biosensors and Bioelectronics* **41**, 103–109 (2013).

4. Lin, Y.-C. *et al.* Graphene annealing: how clean can it be? *Nano letters* **12**, 414–419 (2011).

5. Lin, Y. *et al.* Clean transfer of graphene for isolation and suspension (2011).

6. Sabio, J. *et al.* Electrostatic interactions between graphene layers and their environment. *Physical Review B* **77**, 195409 (2008).
